# Effectiveness of balance training exercise in people with mild to moderate severity Alzheimer's disease: protocol for a randomised trial

**DOI:** 10.1186/1471-2318-9-29

**Published:** 2009-07-16

**Authors:** Keith D Hill, Dina LoGiudice, Nicola T Lautenschlager, Catherine M Said, Karen J Dodd, Plaiwan Suttanon

**Affiliations:** 1 Musculoskeletal Research Centre, Faculty of Health Sciences, La Trobe University, Bundoora, Victoria, 3086 Australia; 2 Allied Health Division, Northern Health, c/o BECC, 1231 Plenty Rd, Bundoora, Victoria 3083, Australia; 3 Preventive and Public Health Division, National Ageing Research Institute, PO Box 2127, The Royal Melbourne Hospital, Parkville, Victoria 3050, Australia; 4 Aged Care Division, Royal Melbourne Hospital (Royal Park Campus), 34–54 Poplar Rd, Parkville, Victoria 3052, Australia; 5 Academic Unit for Psychiatry of Old Age, St Vincent's Health, Department of Psychiatry, The University of Melbourne, Victoria 3010, Australia; 6 School of Psychiatry and Clinical Neurosciences & WA Centre for Health and Ageing, The University of Western Australia, Western Australia, 6009, Australia; 7 Rehabilitation Sciences Research Centre, School of Physiotherapy, University of Melbourne, c/o Royal Talbot Rehabilitation Centre, Kew Victoria 3101, Australia; 8 Physiotherapy Department, Heidelberg Repatriation Hospital, Waterdale Rd, Heidelberg West, Victoria 3081, Australia; 9 School of Physiotherapy, Faculty of Health Sciences, La Trobe University, Bundoora, Victoria 3086, Australia

## Abstract

**Background:**

Balance dysfunction and falls are common problems in later stages of dementia. Exercise is a well-established intervention to reduce falls in cognitively intact older people, although there is limited randomised trial evidence of outcomes in people with dementia. The primary objective of this study is to evaluate whether a home-based balance exercise programme improves balance performance in people with mild to moderate severity Alzheimer's disease.

**Methods/design:**

Two hundred and fourteen community dwelling participants with mild to moderate severity Alzheimer's disease will be recruited for the randomised controlled trial. A series of laboratory and clinical measures will be used to evaluate balance and mobility performance at baseline. Participants will then be randomized to receive either a balance training home exercise programme (intervention group) from a physiotherapist, or an education, information and support programme from an occupational therapist (control group). Both groups will have six home visits in the six months following baseline assessment, as well as phone support. All participants will be re-assessed at the completion of the programme (after six months), and again in a further six months to evaluate sustainability of outcomes. The primary outcome measures will be the Limits of Stability (a force platform measure of balance) and the Step Test (a clinical measure of balance). Secondary outcomes include other balance and mobility measures, number of falls and falls risk measures, cognitive and behavioural measures, and carer burden and quality of life measures. Assessors will be blind to group allocation.

Longitudinal change in balance performance will be evaluated in a sub-study, in which the first 64 participants of the control group with mild to moderate severity Alzheimer's disease, and 64 age and gender matched healthy participants will be re-assessed on all measures at initial assessment, and then at 6, 12, 18 and 24 months.

**Discussion:**

By introducing a balance programme at an early stage of the dementia pathway, when participants are more likely capable of safe and active participation in balance training, there is potential that balance performance will be improved as dementia progresses, which may reduce the high falls risk at this later stage. If successful, this approach has the potential for widespread application through community based services for people with mild to moderate severity Alzheimer's disease.

**Trial registration:**

The protocol for this study is registered with the Australian New Zealand Clinical Trials Registry (ACTRN12608000040369).

## Background

Dementia is a major worldwide health problem among older people. It is one of the most burdensome conditions, and is a leading cause of disability and mortality in later life [1]. The prevalence of dementia is estimated to be 1.5% of people aged 65 years, and prevalence becomes more common with increasing age, reaching a level of 22% in people older than 85 years [2]. The global prevalence of dementia is forecast to double every 20 years, to 42.3 million in 2020 and to 81.1 million people in 2040 [3]. It is estimated that there will be more than one million people with dementia in the UK by 2025 [4]. The number of people with dementia is expected to triple in the United States by 2040 [5], and in Australia by 2050 [6]. Alzheimer's disease is the most common type of dementia, accounting for 50–70% of all dementias [7,8].

Falls are another major public health issue for the older population, with 30% percent of people aged 65 and over falling once or more each year [9,10]. Even higher falls rates have been reported for people with dementia, with around 42% of a community sample with mild-moderate dementia, and 60% of people with dementia in residential care falling at least once each year [11,12]. Decrease in balance performance is a strong indicator of falls in older people [13,14]. Greater balance and gait disturbances have been found in people with dementia when compared with older people in general [15,16], and these have been shown to occur in relatively early stages of the dementia pathway [17]. These declines in balance and motor performance may explain the increased incidence of falls in people with dementia, and have been shown to be a predictive factor for people with dementia needing permanent skilled nursing facility admission [18]. Increased carer strain has also been reported following a fall by care recipients [19].

There has been growing interest in the potential for improved outcomes associated with general exercise for people with cognitive impairment or dementia [20,21], and a number of different exercise approaches have been trialled. Exercise programmes targeting balance performance have been shown to improve balance and reduce falls in a range of community dwelling samples of older people [22-24], but none of these have included people with dementia [25]. While exercise programmes have been shown to be feasible for people with dementia [26,27], even at relatively advanced stages [28,29], these programmes usually primarily incorporate cardiovascular type exercises such as walking (or simple exercises for strengthening and flexibility). To reduce falls in older people, exercise programmes need to safely incorporate a challenge to the balance system [22-24], which requires substantially more complex movement combinations than cardiovascular or strength training exercise programmes. People with dementia are less likely to be able to successfully participate in these more complex movements at later stages of disease progression. Therefore, there is merit in exploring the feasibility and outcomes of balance training exercise programmes for people with mild to moderate severity dementia. Studies in older people living in the community who do not have dementia have demonstrated that improved balance performance from a balance exercise programme can be maintained for periods up to two years [30]. By introducing a balance training programme for people at mild to moderate levels of severity of dementia, it is anticipated that improved balance related outcomes may similarly persist well beyond the period of active engagement in the formal exercise programme. It is also possible that if this type of exercise programme becomes part of routines in earlier stages of the dementia pathway, that these exercises might be able to be continued as dementia progresses, rather than trying to introduce new tasks at later stages.

Given the high proportion of people with dementia who have a diagnosis of Alzheimer's disease, and varying symptoms and progression between people with different types of dementia [31], this study will focus recruitment on people with a diagnosis of Alzheimer's disease. To date, no study has evaluated a balance exercise programme designed to improve balance performance, specifically targeting community dwelling people with mild to moderate Alzheimer's disease.

The primary aim of the present project is to implement and evaluate the effectiveness of an individually tailored, home based exercise programme in improving balance and mobility performance in people with mild to moderate severity Alzheimer's disease. A sub-study will be run concurrently, with the aim of evaluating difference in rate of change in balance performance between people with Alzheimer's disease compared to healthy age and gender matched older people.

## Methods/design

### Study Design

**Figure 1:**
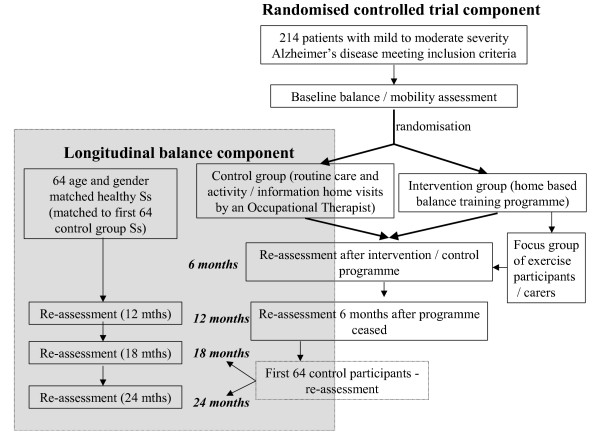
**Study design**.

The primary study is a single blind Randomised Controlled Trial (Figure 1). The CONSORT statement has been used as a framework for development of the methodology for this project. The sub-study is a longitudinal cohort study.

### Participants Criteria

#### Participants with Alzheimer's disease

Participants will be included in the primary study if they have all of the following criteria: (i) a diagnosis of Alzheimer's disease; (ii) mild to moderate dementia (Clinical Dementia Rating Scale score of 0.5–2.0) [32]; (iii) independent mobility (walks outdoors with no more support than a single point stick); (iv) living in the community (ie not living in residential care); and (v) no other major neurological history (e.g. stroke with unilateral or bilateral paresis, multiple sclerosis) or orthopaedic history that impacts on functional mobility.

Participants will be excluded if they have a dementia diagnosis other than Alzheimer's disease; severe dementia (a Clinical Dementia Rating Scale of more than 2.0), limited mobility (doesn't walk away from home, or needs more support than a single point stick to walk outdoors); or presence of clinically significant aphasia (patient needs to be able to understand instructions in the exercise arm of the intervention).

Diagnosis of Alzheimer's disease will be confirmed through records from specialist Memory Clinics or by the treating general practitioner. Memory Clinics are multidisciplinary services established to provide early diagnosis and service provision information for patients with cognitive impairment and dementia and their families [33].

#### Healthy group (longitudinal sub-study)

Sixty-four healthy participants, age and gender matched to the first 64 people with Alzheimer's disease in the control group of the primary study, will be recruited from community advertisements such as newsletters and existing volunteer databases (ie the National Ageing Research Institute database). These participants will have no cognitive impairment (MMSE score ≥ 24) and no serious neurological or orthopaedic condition that impacts on balance and mobility performance, and will be community ambulant (regularly walks independently away from home).

### Measurements and Procedures

At baseline and at subsequent assessment occasions (Figure 1), the following measures will be used:

#### Measures of cognitive impairment and behavioural disturbances include

(i) Mini Mental State Examination (MMSE) [34], a widely used instrument for screening cognitive impairment. It comprises 11 items covering orientation, registration, attention and calculation, recall, language, a 3 step praxis item, and graphic copy of a geometric design.

(ii) Frontal Assessment Battery (FAB) [35], a short cognitive and behavioural battery to assess frontal lobe functions. The FAB consists of 6 subtests exploring conceptualization, mental flexibility, motor programming, sensitivity to interference, inhibitory control, and environmental autonomy. This test has been shown to distinguish frontotemporal dementia from Alzheimer's dementia [36].

(iii) Neuropsychiatric Inventory (NPI) [37], a screening questionnaire to assess 10 behavioural disturbances occurring in people with dementia, including delusions, hallucinations, agitation/aggression, dysphoria, anxiety, euphoria, apathy, disinhibition, irritability/lability, and aberrant motor activity. The NPI will be obtained from a caregiver familiar with the participant's behaviour.

#### Measures of balance and mobility performance

The following **laboratory measures **from the NeuroCom Balance Master™ long plate *(NeuroCom Balance Master™ Operator V3: CopyRight^©^2007) *will be used:

(i) Modified Clinical Test of Sensory Interaction of Balance (mCTSIB), the amount of postural sway is measured under four sensory conditions (eyes open and eyes closed, on a firm and a foam surface); (ii) Limits of Stability (LOS), a measure of the ability to weight shift forwards, backwards, to the right and the left in standing; (iii) stability during walking and turning; and (iv) stability during sit to stand. This test battery has been shown to have moderate to high test-retest reliability in assessing balance dysfunction in healthy older people [38] and clinical groups (eg stroke, Limits of Stability measures, ICC>0.84) [39] and to be sensitive to be able identify patients who have fallen and to be able to predict multiple falls [40].

A number of **clinical measures **of balance and mobility will also be used. These measures are simple and quick tests routinely used in clinical practice and research:

(i) Functional Reach (FR) test [41], a test of dynamic standing balance. The participant stands next to a wall with their feet 10 cm apart and dominant arm raised to 90 degrees. They are then asked to reach as far forward as possible without overbalancing and the distance of additional reach is recorded (cm).

(ii) Step Test [42], a test of dynamic standing balance. The number of times the participant steps one foot fully on and then off a 7.5 cm block step in 15 seconds is recorded. Each leg is tested separately, and worst side performance will be used for data analysis.

(iii) Timed Chair Stands [43]- global leg muscle strength will be measured by timing speed of standing up/sitting down five times from a 45 cm high chair, without using arms.

(iv) Timed Up and Go (TUG) test [44]-the participant is timed standing up from a standard chair, walking 3 metres with their usual speed, then returning to sit again in the chair (seconds). This task will be reassessed under dual task conditions, with a secondary cognitive task (counting backwards by 3's while performing the TUG), and with a secondary motor task (carrying a full cup of water while performing the TUG) [45].

#### Measures of falls and falls risk include

(i) the number of falls in the preceding 12 months will be collected from the participant or carer, based on retrospective recall; (ii) the Falls Risk for Older People (FROP-Com) [46], a detailed falls risk assessment tool evaluating 13 risk factors; and (iii) Physiological Profile Assessment (PPA) [47] abbreviated assessment, evaluating standing balance, hand reaction time, knee joint proprioception, visual contrast sensitivity and quadriceps muscle strength. The FROP-Com and the PPA have been shown to have good retest reliability (FROP-Com intra-rater and inter-tester reliability were 0.93 and 0.81 respectively, and the PPA retest reliability ranged from 0.50 to 0.97), and the FROP-Com to have moderate capacity to predict falls (sensitivity 71% and specificity 56%) [46,47].

#### Caregiver burden and quality of life will be assessed using

(i) the Zarit Carer Burden Scale [48]; and (ii) the Assessment of Quality of Life (AQoL) [49].

### Randomization

After baseline assessment, participants will be randomly allocated into the exercise or control groups. Randomization will be by computer generated random numbers table, with group allocation on a folded piece of paper in an opaque envelope, packed by a staff member independent of the project team. A research assistant independent of the assessors will identify group allocation, and communicate with the participant and their carer as to which group they are in, and make arrangements for the next stage of the project. In this way, assessors will be blind to the group allocation-randomization process at the time of the baseline assessment.

### Intervention Group and Control Group Activities

**The control group **will continue with "usual care" for the subsequent six months without interruption, including all activities and service use recommended by treating health professionals. In addition, participants in the control group will receive a home programme involving six home visits for the six month period by an Occupational Therapist, to provide an information/education and support programme. Topics to be covered in the programme include issues around diagnosis; community services; occupational performance (including issues around grief and losses); and environmental safety and aids. The programme will avoid any messages encouraging any change in activities that may influence balance and mobility performance. Control participants will receive phone calls from the Occupational Therapist to follow up on issues raised in the home visits between home visits.

**The intervention group **will participate in a tailored (individualised) home based balance exercise programme, provided by a physiotherapist. This exercise programme is based on an existing home exercise programme (the Otago programme, ), which has been shown to improve balance and reduce falls in older people without cognitive impairment in the community setting [50-52]. The programme has been modified to provide increased support and visits in the early stages of exercising. Appropriate exercises from the Otago programme will be selected by the physiotherapist, and be tailored to balance and mobility problems identified from the assessments. Participants will receive six home visits during the six month duration (two visits in the first month after the baseline assessment, two further visits in the second month, then two more home visits four to six weeks apart for the remaining times). An exercise booklet will be provided by the physiotherapist to each participant or carer to give illustrations and instructions for the participant to continue the exercise programme at least five times a week. In between visits, the physiotherapist will contact participants by telephone (five phone calls during the six month duration). Adherence to the programme will be monitored by daily completion of an exercise diary. All participants will also continue to receive 'usual care'.

### Follow Up Assessments

Six months after the initial assessment all participants will have all baseline measures repeated by an assessor blind to group allocation. Measures will be repeated six months later (ie 12 months after initial assessment) to determine sustained benefits from the programme.

### Focus Groups

A randomly selected sub-sample of 20 participants from the exercise programme and their caregivers will be invited to participate in focus groups to discuss issues about falls, balance problems, positive and negative aspects of the exercise programme, and other relevant factors influencing participation and outcomes. Data from the focus groups will be recorded, transcribed, and thematically analysed to provide qualitative data informing future recommendations.

### Safety

All assessments will be undertaken by an experienced physiotherapist, providing close supervision during all tasks. In addition, a safety harness will be used for several of the NeuroCom Balance Master™ assessments, which will allow the participant freedom of movement to elicit balance reactions, but will prevent them from falling in the case of overbalancing. The home exercise programme is based on a home exercise programme that has been shown in a number of studies of older people with increased falls risk to be safely implemented, and to reduce falls [50,52]. The frequency of home visits in this study is slightly increased on the successful "Otago" programme (4 visits), aiming to maximise safety with increased visits particularly in the first 6–8 weeks of the programme. The personalized balance exercise programme will be tailored to each individual by an experienced physiotherapist. The exercise prescription and implementation will take into account participant safety.

### Ethical Considerations

Participants will be reassured that their decision as to whether or not they participate in the study is voluntary, and that participation will not influence their usual treatment. Written informed consent will be obtained. If the person with Alzheimer's disease is unable to provide informed consent (as determined by CDAMS records or general practitioner records) to participate (or to continue participation), their next of kin or their caregivers (person responsible) will be asked to provide written consent. The name and contact details of all participants and any information obtained in connection with this research that can identify individual participants will remain confidential and will be used only for the purpose of the research. In any publication, information will be provided in such a way that no individual participant can be identified. All records collected for this research will be kept in a secure place. All records will be archived and retained for a minimum of seven years according to the ethics protocol. At the end of this period, paper-based records will be shredded, and electronically stored data will be erased.

Ethics approval has been obtained from the Melbourne Health Human Research Ethics Committees (HREC 2008.004/Approval Date: 16-Apr-08), and the University Human Ethics Committee, La Trobe University (Application No. 08-011/Approval Date: 30-Jul-08).

### Primary Outcome Measures

Two primary outcome measures have been selected: Limits of Stability (LOS) test (Maximum Excursion measure) (Neurocom Balance Master™) and the Step Test. All other measures will be considered as secondary outcomes. The LOS Maximum Excursion measure is a dynamic balance measure which has been shown to be a sensitive measure of balance impairment, and to be responsive to exercise interventions in older populations [38,40,53]. The Step Test is a simple clinical balance measure which is routinely used in clinical practice and research in the field of balance and falls prevention. It has high reliability [42] and is sensitive to change in balance performance in older people with clinical conditions such as Parkinson's disease [54,55] and stroke patients [42,56], and is responsive to change following an exercise intervention [57].

### Sub-study – Longitudinal Change in Balance Performance

The first 64 participants recruited for the primary study and randomised to the control group will undergo complete assessments as described in the primary study, but also have further complete assessments at 18 months and 24 months following the initial assessment. Sixty four age and gender matched healthy participants will also be assessed on the full balance assessment battery at 0, 6, 12, 18 and 24 months for comparison of the rate of change in balance performance in the sample with mild to moderate severity Alzheimer's disease.

### Statistical Analysis

Intention to treat analysis will be used. Balance performance on the two primary outcome measures and the secondary outcome measures will be made using repeated measures ANOVA between the two groups, with variables differing between groups at baseline being used as covariates. Multivariate logistic regression will be used to evaluate factors associated with improved outcomes, including level of adherence to the exercise programme. For the longitudinal sub-study, independent sample *t*-tests will be used to determine differences in balance performance between both groups at each time point.

### Sample Size

Power analysis was based on data from a recent study of community dwelling older people with mild balance impairment (approximately 15–25% impairment compared to healthy controls), which is in the approximate range of impairment anticipated in our sample with mild-moderate severity Alzheimer's disease. Using data from one of the main force platform measures of balance (Limits of Stability, Maximum Excursion, mean score 71.9; SD 14.3) and a clinical balance measure (Step Test, mean score 14, SD 3.6), power calculations were performed to determine the required sample size to demonstrate a 10% improvement, at 80% power and alpha = 0.05. Using these two tests as primary outcome measures for evaluating the effectiveness of the balance retraining exercise programme, a sample size of 80 per group will be required (160 overall). Assuming a loss to follow up of 25%, a sample of 214 will be required for the main study (RCT study). For the longitudinal sub-study, based on the estimated magnitude of balance impairment (15–25%) relative to healthy age matched controls from previous studies, a sample size of 42 will be sufficient to demonstrate group differences (healthy vs Alzheimer's disease group) on the Limits of Stability Maximum Excursion outcome. Allowing for 20% dropout in this group/year over the 2 year follow-up period for this group, 64 healthy age and gender matched controls (matched to the first 64 participants in the control group) will be required for the longitudinal follow-up component of the study. This sample size will be sufficient to identify significant decline in performance over a two year follow-up period (power 0.8, alpha 0.05, single sided analysis, assuming 7% decline in balance performance/year in the Alzheimer's disease group). Previous studies have shown a change of 1–2% per year on balance and mobility measures for healthy older people [58], and over 10% decline in Activities of Daily Living (including locomotion) in six months in people with moderate severity dementia [59].

### Missing data

To evaluate participant dropout bias, testing for missing at random (MAR) data will be performed. If the missing values are missing at random, the procedures of maximum likelihood (ML) [60] and multiple imputation (MI) [61] will be used for intention-to-treat analyses. In addition, the results from these two intention-to-treat analysis will be compared, as well as the results analyzed with dropped data to determine what difference the three approaches may cause.

## Discussion

Considerable research evidence exists to suggest that a range of single and multiple falls prevention interventions may reduce falls in the community setting [25,62], and exercise is considered to be an effective and essential part of fall prevention programmes [22,23]. In addition, a recent meta-analysis of almost 50 randomised trials investigating exercise interventions to reduce falls in older people has concluded that a focus on balance and lower muscle strength training are key contributing factors to the success of exercise interventions [24]. Despite cognitive impairment being an independent risk factor for falls, most community-based falls prevention studies have excluded participants with cognitive impairments such as Alzheimer's disease. In the few studies that have included participants with Alzheimer's disease, the focus has been on problems of balance impairment, falls and falls injuries at later stages of disease progression, or among those with Alzheimer's disease living in residential facilities [63]. A clear gap in the current research relates to whether there is potential to implement a balance exercise programme at the earlier stages of the disease process in community dwelling people with Alzheimer's disease – particularly when these people still have capacity to safely and actively engage in this type of intervention.

Given (i) the high risk of falls associated with advanced Alzheimer's disease, (ii) the negative impact of balance problems and falls on ongoing care within the home environment, and (iii) the increased risk of hospitalisation or admission to a residential care facility after a fall, this project has the potential for wide applicability within this target group. Specifically, if it could be shown that balance can be maintained or improved at the earlier stages of Alzheimer's disease, this improved balance capacity may possibly reduce fall risks at later stages of the dementia pathway. Furthermore, from a health service perspective, if the approach in the proposed study were successful, it would support a case for balance screening and the provision of a balance training exercise programme as a routine component of early management for people diagnosed with Alzheimer's disease. Finally, delaying onset of balance dysfunction by several years may also have an effect of reducing disability-adjusted life years lost associated with Alzheimer's disease.

## Competing interests

The authors declare that they have no competing interests.

## Authors' contributions

KH conceived of the study, participated in its design and helped draft and revise the manuscript; DL, NL, CS, KD and PS all participated in the study design and helped draft and revise the manuscript. All authors read and approved the final manuscript.

## Pre-publication history

The pre-publication history for this paper can be accessed here:


